# The impact of COVID-19 on mental health in the Hispanic Caribbean region

**DOI:** 10.1017/S1041610220000848

**Published:** 2020-05-08

**Authors:** Jorge J. Llibre-Guerra, Ivonne Z. Jiménez-Velázquez, Juan J. Llibre-Rodriguez, Daisy Acosta

**Affiliations:** 1 Finlay-Albarrán Medicine Faculty, Universidad de Ciencias Médicas, La Havana, Cuba; 2 Department of Internal Medicine, Universidad de Puerto Rico, Medical Sciences Campus, San Juan, Puerto Rico; 3 Department of Internal Medicine, Universidad Nacional Pedro Henriquez Urena, Santo Domingo, Dominican Republic

The recent global severe acute respiratory syndrome coronavirus 2 pandemic will leave its shadow over mental health in our society, especially among the most vulnerable such as elderly populations and those living with mental health disorders, including Alzheimer’s disease (AD) and related dementias. Cognitive impairment and/or dementia itself does not increase the risk for coronavirus disease 2019 (COVID-19). However, people living with cognitive impairment might have difficulties understanding public health information or remembering safeguard procedures (Wang *et al.*, 2020). New measures taken at country level to prevent the spread of the disease are also likely to increase or worsen other mental-health-related disorders like anxiety, depression, substance abuse, post-traumatic stress disorder, and domestic violence (Galea *et al.*, 2020). In early March 2020 Cuba, Dominican Republic and Puerto Rico reported their first COVID-19 cases, and various strategies are underway to tackle with the raising number of cases. The double hit of COVID-19 in elderly populations and among those with comorbid conditions has raised significant concerns in the Caribbean region. In light of these concerns, health care providers and caregivers should pay extra attention to those in most vulnerable situations. In addition, due to the potential impact of COVID-19 on mental health in the elderly population living in the Caribbean area, immediate efforts focused on prevention and early detection of mental health disorders related with the outbreak are required. On this commentary, we examined current situation and impact of COVID-19 on mental health in the Caribbean Hispanic region (Cuba, Dominican Republic, and Puerto Rico). Furthermore, we provide recommendations to health care providers and caregivers to better cope and manage the impact of COVID-19 in our region.

## Aging and mental health in the Hispanic Caribbean in the context of COVID-19

The novel COVID-19 has highlighted the vulnerability of aging populations to emerging diseases, recent data indicates that this virus is of particular risk for older persons, especially those with multimorbidity (Lai *et al.*, 2020). The Caribbean Hispanic represent 57.6% of the Caribbean population (Cuba = 11,326,616, Dominican Republic = 10,847,910, Puerto Rico = 3,193,694) (Quashie *et al.*, 2018). The Caribbean region might be disproportionally affected by the COVID-19 due to a variety of social factors including high proportion of older persons, chronic levels of poverty, income insecurity, and fragile economies that rely heavily on tourism (Quashie *et al.*, 2018). Moreover, in many communities, people live together in close quarters and with extended families which makes social and physical distancing, a critical prevention strategy, more difficult. Among those factors, the aging population and the high prevalence of mental health disorders, including dementia, will pose the higher challenges. During the last decade, the Hispanic Caribbean islands experimented an accelerated demographic aging, due to improved health care standards and high rates of emigration by young adults combined with significant rates of returning nationals at retirement age. At the same time, age-related non-communicable diseases, including mental-health-related disorders, are reported at high prevalence and incidence. For example, according to epidemiological studies in the region, using the same methodology in all three countries, the prevalence of dementia in individuals 65 years and older is approximately 11.7% in Puerto Rico, 11.6% in the Dominican Republic, and 10.8% in Cuba (Rodriguez *et al.*, 2008), being significantly higher than in similar countries in Latin America (Rodriguez *et al.*, 2008). Furthermore, dementia and mental health disorders in general are among the major causes of disability and dependency in older people, representing one of the most serious medical and social issues confronted by Caribbean health systems. In addition, dementia and other mental health disorders overwhelmingly impact not only the people who have it, but also their caregivers, families, and society in general. Depression affects between 10% and 20% of older people and is frequently a comorbidity with anxiety disorders (Rodriguez *et al.*, 2008; Guerra *et al.*, 2016) Therefore, it is expected that the effect of the COVID-19 in the region will have a higher impact on our elderly population and among those with mental health disorders.

The COVID-19 outbreak started in the region in early March, the first cases were reported in the Dominican Republic (March 1, 2020), followed by Cuba (March 11, 2020), and Puerto Rico (March 17, 2020). To date (April 29, 2020), according to local governments reports, more than 86,791 COVID-19 tests have been performed (Cuba = 47,347; Dominican Republic = 26,981; Puerto Rico = 12,463), and 9,873 have been confirmed positive (Cuba = 1,501; Dominican Republic = 6,972; Puerto Rico = 1,400). Cuba reports the lowest number of confirmed cases with 126.9 cases per million people, followed by Puerto Rico and Dominican Republic with 489.4 and 591.5 cases per million people, respectively. The total number of deaths has risen to 456 (Cuba = 61; Dominican Republic = 301; Puerto Rico = 94). The COVID-19 fatality rate (confirmed cases vs. confirmed deaths) has been higher in Puerto Rico (6.1%), followed by Dominican Republic (4.5%) and Cuba (4.0%). The overall fatality rate within the region (4.9%) is lower than the average reported in Europe (11.6%) and similar to the average reported in North America (5.8%) and in other South American countries (4.8%).

Early measures to avoid the spread of the disease have been implemented by local governments since mid-March 2020. A general overview of the measures carry out within the region is provided below.

On March 13, Puerto Rico closed all public schools, and several universities followed canceling in-person classes and switching to remote education. On the same day, Puerto Rico imposed people’s temperature check at all ports of entry. Stay-at-home orders and social distancing guidelines were in place in Puerto Rico by March 15. People could only go out to purchase essential items or obtain essential services from 5:00 AM to 9:00 PM. All businesses, with the exception of grocery stores, supermarkets, gas stations, pharmacies, and medical providers, were required to close.

Dominican Republic declared a state of emergency on March 17, and country authorities announced a series of measures to try and stop the spread of the virus, including the closure of all borders, and suspension of all schools and non-essential commercial businesses. To protect those most at risk, Dominican Republic imposed stay-at-home orders for citizens over 60 years old and those with pre-existing health conditions. Stay-at-home orders were quickly (March 20, 2020) extended to all between 8 PM and 6 AM. On March 26, 2020, the government prolonged the night curfew to 13 hours: from 5 PM to 6 AM.

On March 20, the Cuban Government announced a strict lockdown for vulnerable populations (older adults and people who have serious underlying medical conditions including long-term health conditions, such as diabetes, heart disease, lung disease, autoimmune disease taking immunosuppressive drugs, and people with acquired immunodeficiency syndrome); provisions for home working, employment protection, and social assistance plans were also put in place. On March 24, Cuba closed its borders to international flights and restricted entry to Cuban residents and foreigners residing on the island. As more cases were confirmed, the Cuban Government adjusted its response by the end of March by shutting down public transportation and extending stay-at-home order to the general population (not including essential workers). Rather than opting for mitigation, Cuba has also bet on its primary care system and added a containment plan (prevention and control). Medical students were mobilized nationwide for door-to-door surveys to proactively detect coronavirus cases in the population; possible cases identified through a general screening questionnaire are followed by a community doctor and referred to COVID-19 testing centers. After testing, if confirmed positive, cases will need to stay in isolations facilities. Furthermore, contact tracing and isolation procedures have been imposed to all confirmed cases. If Cuba’s community screening, contact tracing and testing regime get the disease under control, its experience might offer lessons for controlling the pandemic to the rest of the region.

Social and physical distancing measures, taken throughout the region by end of March 2020, are likely to reduce transmission and delay the peak of cases. However, social isolation and loneliness will also have a toll on those at risk for cognitive decline and in our elderly populations by increasing the risk for affective disorders, especially in the most vulnerable groups (Gerst-Emerson and Jayawardhana, 2015).

Physical distancing has precluded our communities from engaging in regular exercise that promotes physical and mental health, and from essential social contact. According to regional statistics, more than 900,000 older adults will not be able to participate in government-run programs aimed to facilitate physical and mental health. The reduction of mobility and not being able to go to social and cultural activities will also have a direct impact on their physical and mental well-being (Santini *et al.*, 2020).

Furthermore, patients with Alzheimer dementia and other dementia disorders are at higher risk to suffer from social isolation and the related stress due to the pandemic. Recent studies have shown lower stress coping ability in AD individuals specially in those with higher burden of tau pathology (Arenaza-Urquijo *et al.*, 2020). People with dementia will face difficulties adjusting to changes in their routine caused by social isolation and restrictions on leaving their homes. This situation may produce more disorientation, sleep disorders, and behavioral changes, such as increased anxiety and agitation.

The novel COVID-19 will also have a significant impact on families caring for someone with dementia or other mental health disorders. Due to the closedown of local supporting services, caregivers have experienced increased physical and emotional overload. As a result, since the beginning of the pandemic, we have observed an increased report on behavioral changes usually related to the alteration in the care arrangements and daily routine.

Finally, it is worth mentioning that by the time the first cases were reported the region was still recovering from major natural disasters, such as Hurricane Maria (September 2017), and the 2019–2020 earthquakes in Puerto Rico. Natural disasters have already put a high burden on our economies and population health. Mental health disorders related to these natural disasters have been widely reported and are likely to be exacerbated by the new pandemic.

## Opportunities for success and recommendations during the COVID-19 outbreak

The Caribbean Islands may have powerful advantages in mounting a successful response to the pandemic. During the last decade, the Caribbean region has faced several epidemics including Dengue, Zika, and Chikungunya, and their experience may prove to be an advantage. Despite previous experiences, the highly transmissible rate and unique characteristics of the novel COVID-19 will require a coordinated response at all levels. Mental health services are posed to offer immediate and long-term actions. In this commentary, we urge policy makers in the Caribbean region to work with researchers and take decisive actions toward the prevention of the direct and indirect effects of the novel COVID-19 on mental health. With the pandemic coming to its peak in the Caribbean region, immediate actions are required. Furthermore, at some point, the acute phase of the pandemic will end and local health care systems should be prepared for the long-term mental health effects of the pandemic in society. Below we provide general recommendations tailored to our region.

1. Special attention should be put on long-term care (LTC) facilities. Prevention measures should include families will not be able to visit LTC facilities; LTC should operate with essential personnel only, health care providers and staff on these centers should be screened with rapid test on a daily basis. It is worth noticing that locking down LTC facilities to family visits will increase social isolation, loneliness, and vulnerability to abuse and neglect, leading to depression, weight loss, and disruptive behavior (Gardner *et al.*, 2020). Therefore, LTC should create spaces for online technologies harnessed to provide interaction with family members, and health care providers should monitor changes in behavior and mental state. Institutions with limited access to online technologies could implement more frequent telephone contact with significant others, close family, and friends. Early measures taken in nursing homes in Santo Domingo, which are mostly run by nuns, have proven to be successful, no COVID-19 cases reported to date.
2. Develop 24/7 helplines and other communication services to support patients, families, and caregivers. Some of these services are already in place, including virtual epidemiological screening.
3. Create screening services to monitor anxiety, depression, and other mental health disorders. Monitoring should target vulnerable populations including elderlies with cognitive impairment/dementia and previous psychiatric comorbidities.
4. Mental health clinics should take extra measures to prevent transmission from health care providers and staff to patients, including expanding symptoms-based screening criteria, facilitating testing, and creating flexible sick leave policies in case of respiratory symptoms.
5. Individuals with mental health issues and elderly populations are more prone to COVID19 atypical presentations or are unable to provide an accurate history. Therefore, health care providers and caregivers should be aware of atypical presentation (Tay and Harwood, 2020), including delirium (hypo and hyperactive), sudden change in behavior, fatigue, falls or loss of appetite. Frontline experience also shows that only 20–30% of geriatric patients with this infection present with fever.
6. Caregivers working with mental health patients will need extra support. Possible strategies may include caregiving training and coping strategies tailored to the current situation. Local resources with information for families and caregivers are already available.
7. Caregivers should encourage their loved ones to engage in mentally healthy behaviors, such as reading, playing table/cards games, and other meaningful activities aligned with their cognitive capacities. One important measure to lower the risk of being infected with COVID-19 is to practice social and physical distancing, not meaning social isolation or loneliness. Therefore, caregivers might help their loved ones to access online services and maintain contact with relatives and friends via phone or video calls.
8. At local level, communities’ health care services should optimize opportunities for social connectedness without breaking stay-at-home rules. A possible alternative could include virtual support groups. The Dominican Alzheimer’s Association has implemented weekly online social support groups and caregiver training sessions. In Puerto Rico, online courses are given to Dementia caregivers and relatives by the School of Medicine.
9. Future research will be needed to better understand the short- and long-term psychological impact of the current pandemic in our societies. Therefore, local governments, funders, and international help will be required to support research in these areas.

Finally, as the new pandemic spreads through the Caribbean, our society, governments, and health care systems will need to be resilient and adjust to the so called “New Normal.” All measures needed to protect those at higher risk in our society should be warranted by the support of local governments and policy makers.
